# Periodontal considerations for Glanzmann's thrombasthenic patient

**DOI:** 10.4103/0972-124X.44095

**Published:** 2008

**Authors:** Umesh Yadalam, K Kranti, Hema Seshan

**Affiliations:** 1 *Ex-Postgraduate Student, Department of Periodontics, M.S. Ramaiah Dental College and Hospital, MSRIT Post, Bangalore - 560 054, India*; 2 *Reader, Department of Periodontics, M.S. Ramaiah Dental College and Hospital, MSRIT Post, Bangalore - 560 054, India*; 3 *Professor and HOD, Department of Periodontics, M.S. Ramaiah Dental College and Hospital, MSRIT Post, Bangalore - 560 054, India*

**Keywords:** Medically compromised, periodontal, thrombasthenia

## Abstract

Glanzmann's thrombasthenia (GT) was reported and described as a bleeding diathesis seen in children and characterized by diminished clot retraction. The disorder is caused by a deficiency in the platelet membrane glycoprotein IIb–IIIa complex, with bleeding due to defective platelet hemostatic plug formation. The recurrent features of GT include purpura, epistaxis, gingival hemorrhage, and menorrhagia. GT being an autosomal recessive trait is reported to be especially prevalent in populations where intermarriage is common. Typically, the patients are diagnosed in infancy within the age of five. Though no differences appear to occur based on sex men more frequently present with gingival bleeding. We report the case of a female patient with GT who presented with the chief complaint of gingival bleeding. The patient was given periodontal treatment under platelet transfusion followed by proper oral hygiene instructions. The report discusses periodontal consideration for GT patients.

## INTRODUCTION

Glanzmann's thrombasthenia (GT) was first described in 1918 as a hemorrhagic diathesis characterized by decreased clot formation seen in children with normal platelet counts, normal prothrombin time (PT) and partial thromboplastin time (PTT), a prolonged bleeding time, abnormal clot retraction, and the absence of platelet aggregation *in vitro* in the presence of adenosine diphosphate (ADP), epinephrine, collagen, or thrombin. This rare autosomal recessively transmitted congenital disorder is characterized and considered among the most frequent inherited platelet diseases and reported to be especially prevalent in populations where intermarriage is common.[1] It is followed by characteristic pattern of mucocutaneous bleeding. The severity is variable, even within kindred. The recurrent features seen in GT include purpura, epistaxis, gingival hemorrhage, and menorrhagia. The disorder is caused by deficiency in the platelet membrane glycoprotein (GP) IIb–IIIa complex,[2] with bleeding due to defective platelet hemostatic plug formation.

GT is differentiated according to fibrinogen content of the platelets and clot retraction. Type I GT in which GP IIb–IIIa complex is <5%, clot retraction is absent, and fibrinogen binding is absent or severely deficient. Type II in which GP IIb–IIIa complex is 10–20%, clot retraction is normal or moderately deficient, and fibrinogen binding is present. Variant type in which GP IIb–IIIa complex is >50%, clot retraction is variable, and fibrinogen binding is variable.[1]

## CASE REPORT

**Figure 1:**
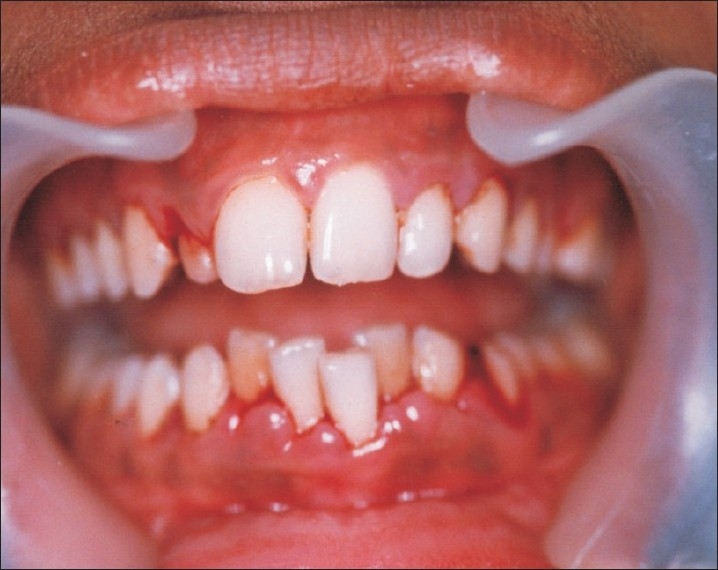
Red, edematous, loss of stippling and knife edge with papillary gingival enlargement, bleeding on probing

A 9-year-old girl, known GT patient, who presented with gum bleeding, was referred from the Pediatrics Department of M.S.R.M.C to the Department of Periodontics M.S.R.D.C. Her medical history revealed that she was born to consanguineous parents. At six months of age she had an episode of epistaxis following trauma and was given blood transfusion for the same. Since then she has had repeated episodes of epistaxis and episodes of bleeding from lips, gums, and tongue due to minor trauma. She had been hospitalized more than 18 times for repeated transfusions to control bleeding. Other clinical manifestations of GT like spontaneous bleeding, menorrhagia, bleeding of purpuric type were absent. On oral examination, her gingival status showed generalized pink colored gingiva. However, it was red, soft, and edematous with stippling and knife-edge lost in mandibular anterior region. Papillary gingival enlargement and bleeding on probing was seen in madibular anterior region. A diagnosis of mild gingivitis with normal periodontal status was made [Figure 1].

Routine laboratory investigations revealed normal hemoglobin (9.6%), platelet count (3,77,000/mm^3^), PT (10.5 seconds), active PTT (29 seconds), and bleeding time (15 seconds). The platelet aggregation test presented no agglutination with epinephrine, ADP, and collagen.

**Figure 2:**
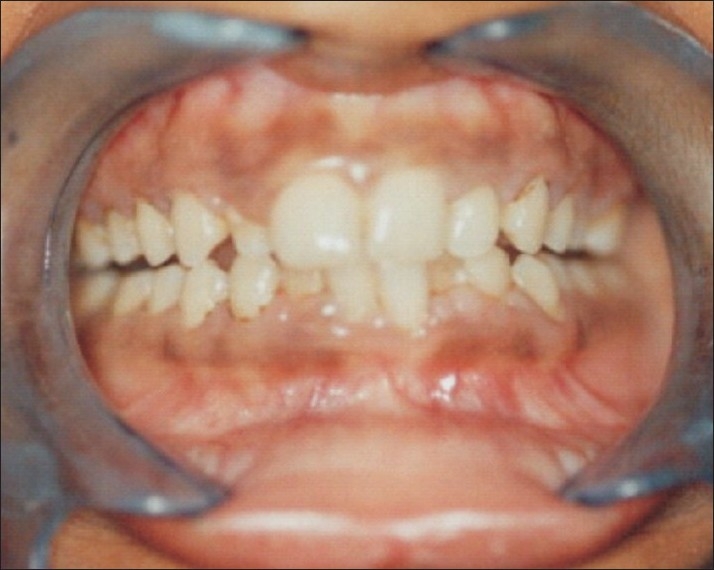
Ultrasonic scaling done and patient recalled after one week

Periodontal treatment (scaling and polishing) under platelet transfusion was given followed by and oral hygiene instructions. The patient was prescribed 0.12% chlorhexidine and tranexamic acid mouth rinses. The therapy was successful with decrease in gum bleeding and the patient returning to *status quo*. Thereafter, she had weekly follow-up for one month and monthly recalls for the next six months [Figure 2].

## DISCUSSION

GT is a familial disorder occurring in both the sexes. Most patients are diagnosed before the age of five. The main problem is the occurrence of hemorrhage whose frequency and severity may decrease with age. However, it is important to anticipate the risk of bleeding and administer prophylactic platelet transfusion even if the patient does not have a history of prior bleeding, because bleeding in thrombasthenia is unpredictable. Previous reports and investigations on the clinical management of the disease present only palliative means of treatment.[3]

It is suggested that any periodontal procedures involving the risk of hemorrhage be done at the time of platelet infusion. If this is not possible the prophylactic methods and transfusion should be performed with utmost care and upon consultation with the patient's hematologist. The treatment modalities should be conservative as far as possible and procedures that would necessitate repeated visits should be avoided. Optimal oral hygiene should be encouraged and a soft-bristle toothbrush prescribed. Preventive therapeutic means are very important in GT patients. Antiplaque agents are beneficial for the maintenance of proper oral hygiene in the postoperative period. A 0.12% chlorhexidine mouth rinse was used in our case and was found to be beneficial in controlling plaque. Although the tooth staining necessitates additional scaling appointments, regular use of ultrasonic scalers and polishing with rubber cups benefits patients without any further risk of hemorrhage.[4]

It would be wise to initiate meticulous oral hygiene immediately in all patients with this disorder. If a thorough medical history is obtained, a hematological pretreatment is sought, precautions are taken, and a hematological pretreatment is conducted prior to the referral to dental office, periodontal treatment can be successfully carried out.

The differential diagnosis in patients with mucocutaneous bleeding, prolonged bleeding times, and normal platelets includes von Willebrands disease and Bernard–Soulier syndrome in addition to Glanzmann's thombasthenia, although it may not be easy to diagnose and differentiate among many bleeding disorders in which severe hemorrhage is seen. A dental and hematological close collaboration therefore is essential for the management of such complicated cases.[5]

## CONCLUSION

If a thorough medical history is obtained, precautions are taken, and a hematological pretreatment is conducted prior to the referral to dental office, periodontal treatment can be successfully carried out.
